# Mechanical inserts for the treatment of faecal incontinence: A systematic review

**DOI:** 10.1080/2090598X.2019.1589776

**Published:** 2019-04-08

**Authors:** Kristen Buono, Bhumy Davé-Heliker

**Affiliations:** Division of Female Pelvic Medicine and Reconstructive Surgery, Department of Obstetrics and Gynecology, University of California Irvine, Orange, CA, USA

**Keywords:** Faecal incontinence, accidental bowel leakage, mechanical insert, vaginal insert/plug, anal insert/plug

## Abstract

**Objective**: To perform a systematic review of the literature to examine original research on the role of mechanical inserts, both vaginal and anal, for the treatment of faecal incontinence (FI).

**Materials and methods**: We searched the PubMed, Cochrane Library, and ClinicalTrials.gov databases for any peer-reviewed original research in English on the role of mechanical inserts for the treatment of FI.

**Results**: We identified 35 unique citations. After title review and exclusion of articles not reporting original research, eight publications were included in the final review: two focused on vaginal inserts and six focused on anal inserts. Limited evidence indicates that both vaginal and anal inserts can be an effective and safe therapeutic option for patients with FI.

**Conclusions**: Data regarding vaginal and anal mechanical inserts for the treatment of FI, albeit limited, suggest that inserts can be included in a discussion of therapeutic options for a patient with FI. Further studies are needed to elucidate long-term usability, efficacy, and safety.

**Abbreviations**: FI: faecal incontinence; (m)ITT: (modified) intention-to-treat

## Introduction

Faecal incontinence (FI), also referred to as accidental bowel leakage, is defined as the involuntary passage of faecal material through the anal canal. It is a debilitating condition that causes a significant impact on a patient’s quality of life, and represents an unmet need in women’s healthcare today. Current epidemiological studies have reported rates of FI in up to 18% of community members and up to 47% of nursing home residents [1–7]. This condition is often stigmatised, which can lead to embarrassment, social isolation, and reluctance to seek treatment from patients who are unaware that help is available.

Conservative treatment options for FI include stool consistency manipulation via dietary modifications, fibre bulking agents, or anti-diarrhoeal medications, as well as pelvic floor exercises with or without biofeedback. Surgical options include the injection of bulking agents, radiofrequency energy sphincter remodelling, sacral neuromodulation, anal sphincteroplasty, artificial bowel sphincter, or magnetic anal sphincter implantation [8–12]. Many of the available treatments for FI have considerable shortcomings with regards to efficacy, morbidity, patient compliance, and cost. Additionally, many therapies are primarily directed at addressing only one aspect of continence, which makes their widespread application difficult. When FI persists despite active treatment, one option to consider is faecal containment via a mechanical insert placed in either the vagina or anus. The purpose of the present review is to better understand the overall efficacy and patient satisfaction of vaginal and anal inserts for the treatment of FI.

## Materials and methods

This systematic review was performed according to the Preferred Reporting for Systematic Reviews and Meta-Analyses (PRISMA) guidelines [13]. PubMed, Cochrane Library, and ClinicalTrials.gov databases were searched to identify peer-reviewed original research on mechanical inserts for the treatment of FI. Search terms included both keywords and official vocabulary for each database, for ‘faecal incontinence’ and either ‘insert’, ‘vaginal insert’, or ‘anal insert’. The databases were searched without any restriction on date of publication. The search included all manuscripts available for search in July 2018. References of the papers included were searched to find additional relevant publications.

The aim of the present review was to evaluate the efficacy of various mechanical inserts for the treatment of FI, including both vaginal and anal inserts. Eligibility for inclusion were studies of any design type reporting original data on vaginal or anal inserts for the treatment of FI. The search was restricted to English-language publications only. Studies were excluded if they presented duplicate data or were defined as review papers; however, the references of review papers were searched to find additional relevant publications. The abstract and titles were screened and full-text copies were retrieved if the study was considered potentially eligible. Data extracted included the type of study, number of patients included, and significant conclusions related to the study aim.

## Results

**Figure 1.**
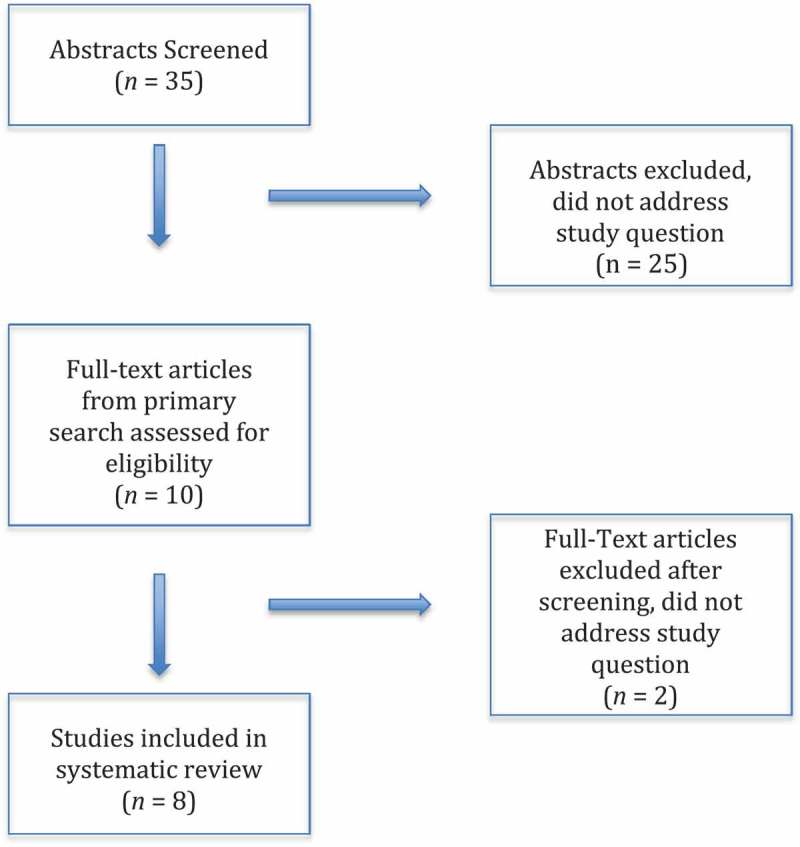
Flow diagram of study selection.

The search identified 35 unique citations and 10 citations met criteria for full-text review. Two publications were excluded because they were review articles. Thus in total, eight studies met the inclusion criteria (Table 1 [14–21], Figure 1). Three of these publications were derived from the PubMed search and five publications were identified through examination of the references of reviewed publications. These studies varied in methodology and in areas of inquiry.

**Table 1.** Summary of studies selected for review.

Study	Insert type	*N*	Population	Study design	Duration	Treatment outcomes	QoL	Discontinuation rate	AEs	Strengths	Weaknesses
Richter et al., 2015 [14]	Eclipse vaginal insert	110 (ITT: 61 PP: 56 3-months: 44)	Adult women with ≥2 FI episodes/week	Prospective effectiveness and safety trial	1 month, 3 months	1 month: 48/61 subjects had >50% FI reduction 39/56 subjects had >75% FI reduction 23/46 subjects had 100% continence 3 months: 38/44 subjects had >50% FI reduction 32/44 subjects had >75% FI reduction 20/44 subjects had 100% continence	-Increased FIQOL -Decreased MMHQ -50/56 were satisfied -54/55 would recommend to a friend	49 subjects (44.5%) did not achieve a successful fit	No serious AEs 18 device-related AEs (primarily pelvic discomfort or vaginal findings on examination)	Multiple validated outcome measures	Small sample, short study duration, lack of placebo group
Varma et al., 2016 [15]	Eclipse vaginal insert	56	Adult women with ≥2 FI episodes/week	Secondary analysis	1 month	15% reduction in liquid bowel movements 28% reduction in faecal urgency 13% reduction in incomplete evacuation	N/A	N/A	N/A		Descriptive analysis
Mortensen and Humphreys, 1991 [16]	Conseal anal insert (3 designs)	10	Adults (8 female, 2 male)	Cross-over pilot trial	3 weeks (each anal insert design for 1 week)	Incontinent defecations: Design #1–18% Design #2–19% Design #3–15% Median duration of use/day: Design #1–12 h Design #2–11 h Design #3–7 h	Easy insertion: Design #1–79% Design #2–82% Design #3–52% Preferred Design: #1–5/9 subjects #2–3/9 subjects #3–1/9 subjects	1 subject (10%)	No serious AEs reported -Persistent discomfort in 2/9 subjects	Cross-over design	Small sample, short study duration
Christiansen and Roed-Petersen, 1993 [17]	Conseal anal insert (Design #1 from prior study)	14	Adults (9 female, 5 male)	Prospective pilot trial	4 weeks	9/14 subjects were continent with the device in place	All 14 subjects reported they felt ‘safe’ and ‘mentally better’ with the device in place	11 subjects dropped out due to discomfort 1 subject (6%) did not follow up for treatment	No serious AEs reported		Small sample, minority of subjects completed the study protocol
Norton and Kamm, 2001 [18]	Conseal anal insert (37 and 45 mm)	20	Adults (16 female, 4 male)	Cross-over pilot trial	4 weeks (each insert for 2 weeks)	10/20 subjects were continent with the device in place No difference in comfort between subjects with intact vs impaired rectal sensory function	4 subjects (20%) reported they would use the insert continuously No difference in comfort between the 2 inserts	9 subjects (45%) dropped out after the first insert due to discomfort	No serious AEs reported	Cross-over design	Small sample, minority of subjects completed the study protocol
Pfrommer et al., 2000 [19]	Conseal anal insert vs EFF-EFF anal insert	38	Children with prior repair of anorectal malformation	Randomised cross-over trial	6 weeks (each insert for 3 weeks)	12/23 subjects endorsed 100% continence with both inserts 61% of patients preferred the Conseal insert vs 22% who preferred the EFF-EFF insert	Preferred insert: Conseal – 14/23 EFF-EFF – 5/23 No preference – 4/23	15 subjects (39%) dropped out due to discomfort	No serious AEs reported	Cross-over design	Small sample, paediatric patient population with prior congenital malformation
Giamundo et al., 2002 [20]	Procon anal insert	18 consented, 7 completed the study protocol	Adults (5 female, 2 male)	Prospective pilot trial	2 weeks	Decreased Cleveland Clinic Florida Incontinence Score (mean 7.5 points)	5 subjects (28%) reported complete satisfaction	11 subjects (61%) dropped out due to hypersensitivity or difficulty operating the device	No serious AEs reported	Validated outcome measure	Small sample, minority of subjects completed the study protocol
Lukacz et al., 2015 [21]	Renew anal insert (ITT: 91 mITT: 85 PP: 73)	97	Adults with ≥ 1 FI episode/week (82 female, 9 male)	Prospective single-arm study	12 weeks	1 month: 66/85 subjects had >50% FI reduction 3 months: 56/73 subjects had >50% FI reduction FI frequency reduced from 0.9 to 0.2 FI episodes/day 26% reduction in FI frequency from baseline after subjects discontinued treatment for 4 weeks	78% of subjects who completed treatment were very or extremely satisfied	12 subjects dropped out if they did not meet eligibility, found the protocol too demanding, or repeatedly lost the insert (12%)	No serious AEs reported 3 moderate AEs (faecal urgency, haemorrhoid bleeding)	Return to baseline evaluation, daily bowel diaries	Short study duration, lack of validated questionnaires

AE, adverse event; FIQOL, Fecal Incontinence Quality of Life; MMHQ, Modified Manchester Health Questionnaire; PP, per protocol, QoL, quality of life.

### Vaginal inserts

**Figure 2.**
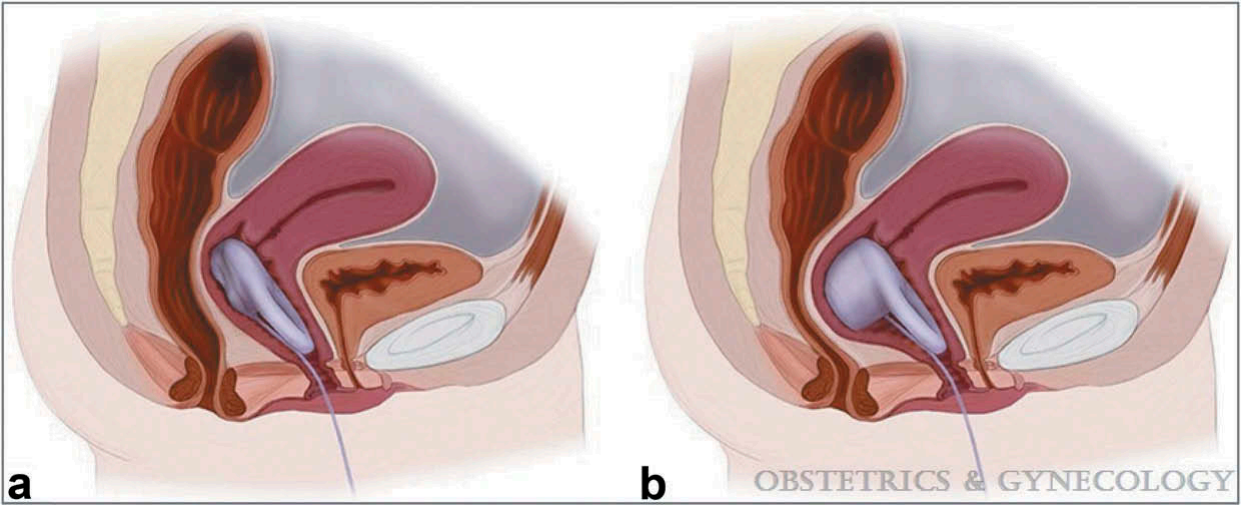
Vaginal bowel-control insert *in situ*. Deflated for defaecation (a) and inflated to prevent accidental passage of stool (b). From Richter et al. [14].

Current data on the role of vaginal inserts for the treatment of FI are limited; two papers are included in the present review. In a prospective, multi-site, open-label effectiveness and safety trial of 110 female patients with a minimum of four FI episodes during a 2 week baseline period, 61 participants (55.5%) achieved a successful fitting of an Eclipse System vaginal insert (Pelvalon Inc., Sunnyvale, CA, USA) [14]. The Eclipse System consists of a silicone-coated stainless steel base vaginal insert and pressure-regulated pump (Figure 2). The vaginal insert has a posteriorly directed dual-layer balloon that provides reversible occlusion of the rectum, which enables the patient to control her own bowel movements. The insert is simply deflated, rather than removed, during defaecation. It is recommended to remove the vaginal insert for cleaning once a week, as well as prior to coitus.

After 1 month, 48 of the 61 (78.7%) participants who had a successful fitting had treatment success, which was defined as a ≥ 50% reduction in FI episodes over a 2-week treatment diary. In all, 85% of the per-protocol study population, who had completed the full 1 month use and the study diaries, had treatment success, whilst 69.6% had at least a 75% reduction in FI episodes. In all, 23 of the 56 (41.1%) per-protocol participants had complete continence after 1 month of use of the vaginal insert. About 90% of participants were satisfied with their experience using the vaginal insert and 98.2% would recommend it to a friend. After the 1-month treatment period, 96% of participants reported that the insert was comfortable (48%) or they could not feel it (48%).

In all, 44 of the 56 participants (78.6%) chose to complete the optional 2-month extended-wear period, which provided 3 months of data. After 3 months, 86.4% of the participants had a 50% improvement, 72.7% had a 75% improvement, and 45.5% had complete continence. The mean (SD) FI episodes recorded at the end of the extended-wear period were 1.7 (2.0) episodes over 2 weeks (compared to 11.6 (9.5) episodes at baseline, *P* < 0.001). There were no serious device-related adverse events during any part of the study. Of the 61 participants who achieved a successful fit and entered the treatment period, 14 participants had 18 device-related mild or moderate adverse events during the 1-month treatment period, the most common being pelvic cramping or discomfort.

A secondary analysis of the 56 participants who completed the study diaries showed that the total number of bowel movements per patient decreased from 20.9 to 15.3 on a 2-week diary after 1 month of use of the vaginal insert (*P* < 0.001) [15]. Additionally, after 1 month of use of the vaginal insert, the percentage of bowel movements reported as liquid reduced from 36% to 21%, and the percentage of bowel movements associated with urgency reduced from 54% to 26%. The authors suggested that a possible mechanism for the change in stool consistency may be due to dampening of continence-related reflexes or increased water absorption as a result of the physical occlusion of the anal canal. A multi-site, open-label trial evaluating clinical outcomes after 3 and 12 months of continuous use of the Eclipse vaginal insert has recently completed patient enrolment [22], which will provide further information regarding the safety and long-term efficacy of this therapy.

### Anal inserts

The concept of an anal insert for the treatment of FI was first evaluated in a small pilot study of 10 participants [16]. Participants’ trialled three different designs of Conseal anal insert (Coloplast, Humlebæk, Denmark) over 3 weeks, one per week, and kept a daily bowel diary. The three plug types all consisted of a polyurethane sponge wrapped in a water-soluble coat that reduced their size to that of a conventional suppository. Once inside the anus, the water-soluble coat dissolved and the plug expanded to its full size. Participants were instructed to insert the lubricated plug into the anus after defecation and expel it by either pulling on the string or raising the intra-abdominal pressure to expel it.

Nine of the 10 participants completed the 3-week study. One participant withdrew after 2 weeks secondary to discomfort with the insert. The median hours of use for a single anal insert ranged between 7 and 12 h based on the type of anal insert. Two anal insert types were easily inserted as a suppository on 79% and 82% of occasions, respectively, but the third type was only easily inserted on 52% of occasions. The anal insert either slipped out or was removed because of discomfort in 12–20% of occasions. Overall there was no faecal leakage in 83% of cases. At the completion of the study, five participants preferred the type 1 anal insert, three preferred the type 2 anal insert, and one preferred the type 3 anal insert. Pain, discomfort, and bleeding on insertion or removal were rare, and faecal urgency with the insert in place was not specifically reported.

A follow-up study of 14 patients was performed to evaluate the type 1 Conseal anal insert that was preferred by the majority of participants from the previously described study [17]. Nine of 14 patients were continent with the insert in place; however, six participants reported slippage of the anal insert. Ultimately, 10 participants experienced discomfort to a varying degree and 11 participants withdraw from the study before the end of the planned study period. All patients reported that they felt safe whilst using the insert and that they would use it for certain occasions in the future.

A follow-up randomised cross-over trial was conducted to evaluate the efficacy of the Conseal anal insert in patients with various types of FI [18]. Participants were asked to try two sizes of the plug, in random order, each for 2 weeks. Of the 34 patients offered the anal insert, four refused, two failed to attend the first appointment, and eight dropped out immediately after trying the anal insert on one or two occasions (with discomfort as the stated reason in all cases), which resulted in a total of 20 participants who attempted the anal insert for an extended period of time. In all, 11 participants used the larger (45 mm diameter) insert and nine used the smaller (37 mm diameter) insert. Nine of the 20 total participants (16 women and four men) dropped out after trying only one size of the anal insert, refusing to try the second size. In all, 10 of the 20 participants (50%) were continent whilst using the insert and four had greatly improved continence. Interestingly, five of the 11 participants who tolerated the anal insert well enough to complete the study refused to continue using the anal insert due to discomfort or a continual desire to defaecate. Two participants reported that they would use the insert occasionally and the remaining four reported that they would use the insert on a regular basis.

Eight of the 11 participants who completed the study protocol reported complete continence with the anal insert. There was no difference in efficacy or comfort between the two sizes of the anal insert. Those participants with impaired sensory function did not find the anal insert to be more comfortable than participants with normal sensory function, and two of the four participants who chose to continue using the insert had normal sensation. Overall, of the original 34 patients offered the anal insert, only six (18%) participants chose to wear it on a regular or occasional basis. These results were similar to those of the previously described follow-up study, where 71% of participants discontinued use of the anal insert secondary to discomfort [17].

A randomised, cross-over trial was conducted amongst paediatric patients following imperforate anus repair to compare the efficacy of the Conseal anal insert vs the EFF-EFF polyvinyl-alcohol anal insert (Med. SSE-System, Nürnberg, Germany) [19]. Participants in both groups tested each type of anal insert for 3 weeks. Most study participants used the anal insert for 5–9 h on a daily basis, and a minority of participants used the insert infrequently. In all, 15 of the 38 participants (39%) failed to complete the study protocol. Overall, 12 of the 23 participants (52%) who completed the study protocol reported complete continence whilst wearing either anal insert. A non-significant difference in acceptable effectiveness, which was defined as ≤2 episodes of FI over 3 weeks, was seen between products (65% for the Conseal anal insert vs 74% for the EFF-EFF anal insert). At the completion of the study, 61% of participants preferred the Conseal anal insert vs 22% who preferred the EFF-EFF anal insert (*P* < 0.05). In all, 17% of participants reported no difference between the two products. Information regarding whether participants would continue to use the anal inserts after the study concluded was not reported.

An alternative anal insert, the Procon device (AnaTech, El Paso, TX, USA), was first evaluated during a prospective, open-label, pilot study [20]. This particular anal insert consists of a disposable, double lumen, biochemically inert, pliable, cuffed rubber catheter that incorporates an infrared photo-interrupter sensor in the distal tip. About 1 cm proximal to the sensor, the catheter has an inflatable 20-mL capacity cuff, which is similar to that in a bladder catheter. The device is intended for self-insertion. After insertion, the balloon cuff is inflated with 20 mL air and the catheter is gently withdrawn until it encounters the resistance of the levator ani musculature. The catheter is connected to a monitor, which is connected to a pager that can be worn on the patient’s belt. When stool enters the rectum, the photo-interrupter sensor sends a signal to the pager, which alerts the patient of an imminent defaecation. The inflated balloon acts a mechanical barrier to allow the patient to have more time to reach a toilet, thereby conceivable precluding any FI. The balloon is deflated and catheter is withdrawn prior to voluntary defecation, and re-inserted after the rectum is sufficiently emptied.

A total of 18 participants were consented to participate and of these, seven completed the 14-day trial. Eight participants reported the device was too ‘difficult’ to manipulate and three participants experienced hypersensitivity of the sensor so the pager was kept in the ‘off’ position for the majority of the study. Of note, the three participants that experienced hypersensitivity had continuous mucous discharge or watery diarrhoea prior to enrolment in the study. Of the seven participants (five female and two male) who completed the trial, five participants reported complete satisfaction. There were no reported complications.

**Figure 3.**
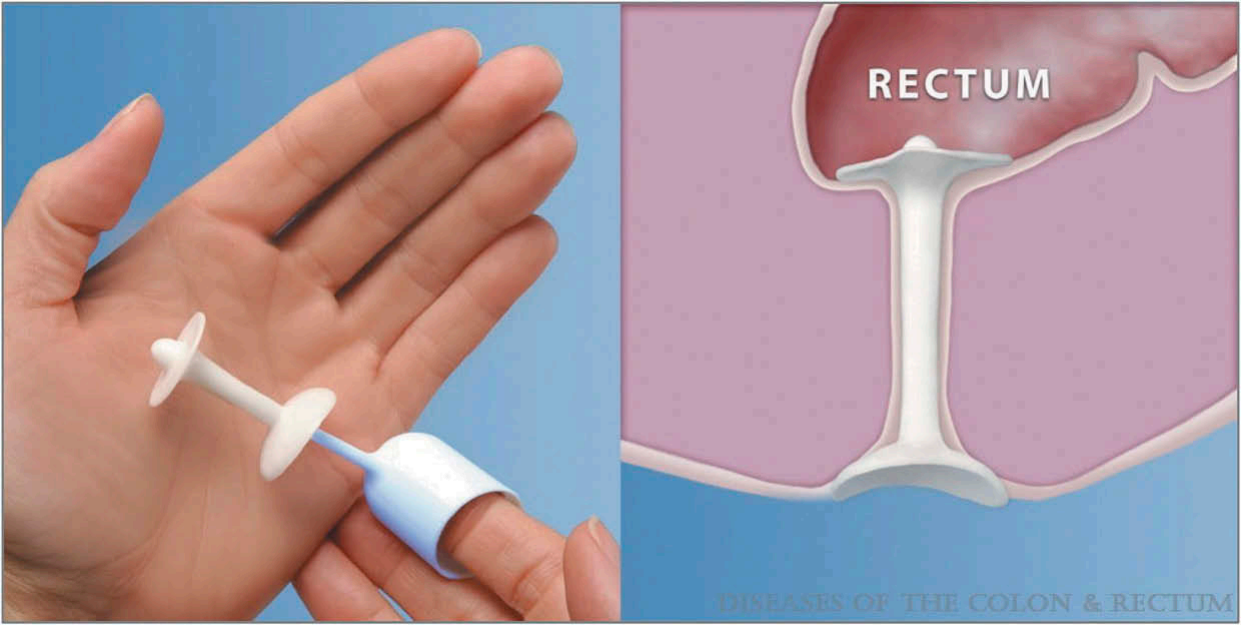
The anal insert device with finger applicator and *in situ*. From Lukacz et al. [21].

Most recently, a multi-site, prospective, nonrandomised, single-arm study of the Renew anal insert (Renew Medical Inc., Foster City, CA, USA) was reported [21]. This device is a single-use, soft silicone anal insert that is self-inserted with the use of a fingertip applicator (Figure 3). The top disk of the insert forms a seal at the top of the anal canal, whilst the stem spans the anal canal and the bottom disk remains outside the anus to help prevent displacement of the insert. It is available in two top disk diameters (22 and 28 mm) and is designed to be self-expelled during voluntary defecation or can be manually removed by pulling on the bottom disk. Participants underwent 4-week baseline evaluation including daily bowel diaries, followed by a 12-week treatment period of continuous device use. The primary outcome of objective success was defined as ≥50% reduction in FI episodes and subjective success was measured by reduction in Cleveland Clinic Florida Fecal Incontinence Score/Wexner severity score.

In all, 97 participants were enrolled, of which 91 remained eligible after the 4-week baseline evaluation (intention-to-treat cohort, ITT). In all, 85 participants completed at least 1 week of treatment (modified ITT cohort, mITT) and 73 participants completed the full 12 weeks of intended therapy (completer cohort). An average of 2.6 inserts was used per day, with 66% of them being expelled during defecation. In all, 62% of participants had objective success in the ITT cohort, whilst this success rate was 78% and 77% in the mITT and completer cohorts, respectively. The median FI frequency was reduced by 82%, from 0.9 FI episodes/day at baseline to 0.2 FI episodes/day at 12 weeks. Regarding timing, there was a 77% reduction in symptoms in the first week of use and a 93% reduction by 4 weeks of use of the anal insert.

There were no serious adverse events and only three moderate adverse events (faecal urgency, soreness, and bleeding haemorrhoids) in two participants during treatment amongst the 91 participants in the ITT cohort. Displacement of the anal insert upward into the anal canal occurred in 24% of participants, but resolved with natural expulsion during defaecation. Overall, 78% of completers were very or extremely satisfied with the anal insert and 91% of them rated the overall experience, comfort, and ease of insertion ≥8 on a 10-point scale (median score 9.5). The authors suggested that the smaller volume of the Renew anal insert that actually resides in the rectum does not stimulate the anal sensory system as much as previously studied anal inserts, which resulted in lower discontinuation rates as reported previously.

## Discussion

FI is a common condition with a profound negative impact on a patient’s quality of life [23]. Unfortunately, due to social stigmatisation and embarrassment, <30% of patients with this condition seek treatment [24]. There are a wide spectrum of treatment options for FI, including pelvic floor exercises with biofeedback, stool consistency management through the use of dietary modifications or pharmacotherapy, mechanical obstruction via vaginal or anal inserts, injection of bulking agents, sacral neuromodulation, radiofrequency energy sphincter reformation, anal sphincteroplasty, artificial bowel sphincter, and magnetic anal sphincter implantation [8–12,14,18,20,21,25]. The morbidity, cost, and long-term efficacy of each therapeutic option is variable and should be considered in the context of each individual patient’s general overall health, degree of FI, and insurance coverage. Mechanical inserts, whether vaginal or anal, are passive obstructing barriers to help prevent FI and provide immediate relief. This treatment option attempts to fill a significant gap in effective therapeutic options with low morbidity. Mechanical inserts have the potential to be used as a stand-alone therapy or be used in conjunction with other conservative therapies including pelvic floor exercises, biofeedback, dietary modifications, or the use of oral medications to manipulate stool consistency.

Expandable and inflatable anal plugs are designed to treat FI by blocking the flow of liquid and solid stool from the rectum. Unfortunately, the adoption of anal inserts has been limited due to previous anal insert designs, which were found to be intolerable or difficult to use by patients [16–20]. The most recent evaluation of the Renew anal insert showed promising clinical application, as 80% of the eligible participants completed 12 weeks of therapy and 78% of those who completed treatment were very or extremely satisfied with the anal insert [21]. However, the primary limitations of this study include the use of a non-validated modification to the FI severity score questionnaire, as well as the lack of a control comparison group, randomisation, blinded assessments, and FI-related quality-of-life measures.

Whilst anal inserts for the treatment of FI have been studied for almost 20 years, a vaginal insert for the treatment of FI has emerged as a new therapeutic option within the last several years. Using the vagina as a potential space to influence the function of a nearby structure has been utilised for the passive management of urinary incontinence for decades [26,27]. The concept of a dynamic vaginal insert that reversibly occludes the rectum to interrupt the passage of stool and is not displaced during defaecation, represents a significant shift in FI management. Overall the patient satisfaction was noted to be almost 90% with the Eclipse vaginal insert and no serious adverse events were reported after 3 months of continuous therapy. The primary limitations of this study were the high rate of unsuccessful fitting (45%), as well as the lack of a control comparison group, randomisation, blinded assessments, and short duration of follow-up. Currently a multi-site, open-label trial evaluating longer-term outcomes is being conducted [22], and will provide a better understanding of the safety and long-term efficacy of this particular therapy.

The present review of mechanical inserts for the containment of FI was limited by the small quantity of eligible studies and participants; furthermore, pooling data in a meaningful way was either impossible or inappropriate. The present data indicate that additional larger-scale randomised comparative studies to other standard-of-care therapies using validated FI-related questionnaires are needed. Further studies should also aim to characterise optimal patient selection with more detailed demographic, patient history, and physiological testing. Whilst the studies evaluating vaginal and anal inserts for the treatment of FI are currently limited, the emerging evidence is promising and suggests that this therapeutic option could be used as either an individual therapy or in conjunction with other conservative treatment options for patients with FI.

## Conclusions

The present systematic review summarises the existing literature on the role of mechanical inserts for the management of FI. Promising early studies suggest that newer models of anal inserts, as well as the first vaginal insert, are effective and satisfactory therapeutic options for patients with FI. Further research is needed to investigate the long-term safety and efficacy of this treatment, as well as compare this therapeutic option to other conservative therapies for FI. In the absence of a significant body of evidence, it is reasonable to include mechanical inserts as a therapeutic choice when counselling a patient with FI about her treatment options.
